# Deep learning–driven fine-resolution finger movement recognition from surface EMG signals

**DOI:** 10.1371/journal.pone.0361050

**Published:** 2026-10-22

**Authors:** Hiam Alquran, Hassan Alawneh, Mohammed Alsalatie, Ateka Khader, Wan Azani Mustafa, Khaled Zayed Alawneh

**Affiliations:** 1 Biomedical Systems and Informatics Engineering Department, Yarmouk University, Irbid, Jordan; 2 Hunter Medical Research Institute, Mark Hughes Foundation Centre for Brain Cancer Research, University of Newcastle, Newcastle, NSW, Australia; 3 The Institute of Biomedical Technology, King Hussein Medical Center, Royal Jordanian Medical Service, Amman, Jordan; 4 The Department of Biomedical Engineering, Jordan University of Science and Technology, Irbid, Jordan; 5 Faculty of Electrical Engineering & Technology, University of Malaysia Perlis, UniCITI Alam Campus, Arau, Perlis, Malaysia; 6 Advanced Computing (AdvCOMP), Centre of Excellence, Universiti Malaysia Perlis (UniMAP), Arau, Perlis, Malaysia; 7 Department of Diagnostic Radiology, Faculty of Medicine, Jordan University of Science and Technology, Irbid, Jordan; Universita Politecnica delle Marche Facolta di Ingegneria, ITALY

## Abstract

Surface electromyography (sEMG) has emerged as an impactful tool for controlling advanced prosthetic devices by detecting and recording muscle electrical activity during motion. This study presents a unique technique for recognizing nine distinct classes of finger movements by utilizing the Ninapro database. To address an underexplored domain of upper-limb prosthetic control, this research targets fine-resolution individual finger movements rather than the broad range of hand gestures. A custom-made one-dimensional convolutional neural network (1D–CNN) was developed and validated using a within-subject evaluation strategy and signals. The proposed model achieved an overall classification accuracy of 90.1%. An in-depth sensitivity/specificity analysis for each finger movement class was administered to gain key insights into current recognition capabilities. The findings presented in this study demonstrate the viability of fine-resolution finger-movement recognition, offering foundational insights that may inform the future development and refinement of control systems in the prosthetic field.

## 1. Introduction

Current advancements in prosthetic technologies reserve the potential to improve upper-limb rehabilitation by offering enhanced motor functionality via electromyography (EMG)-controlled devices. The EMG-controlled devices are designed to restore a more natural and dexterous sense of hand function for individuals characterized by upper limb loss. These devices therefore represent a critically needed advancement in assistive technology for this population [1,2]. Constrained by both mechanical simplicity and a primitive control paradigm, traditional prosthetic devices often fail to replicate complex human motion [3]. EMG signals, designed to reflect the electrical activity of muscles, offer valuable insights for replicating natural hand gestures. However, their application is challenged by the accurate decoding of motor intent due to their non-stationary nature and reported susceptibility to noise from several factors, including muscle fatigue, electrode displacement, and intersubject variability [4].

The rise of artificial intelligence (AI) and machine learning (ML) applications has brought forth an intention to facilitate a paradigmatic shift within EMG-based control systems [5–7]. Deep learning models have demonstrated high proficiency in detecting complex spatiotemporal patterns, facilitating the automatic extraction of discriminative features from raw input data [8–10]. High accuracy, despite signal noise and inter-user variability, is observed in applications of convolutional neural networks (CNNs) and recurrent neural networks (RNNs), thereby propelling recognition systems forward [11,12]. While CNNs have been extensively investigated for sEMG-based pattern recognition, more recent architectures, namely Vision Transformers (ViTs), have also shown promising results in related classification tasks. The potential advantages of utilizing ViTs include capturing both long-range dependencies and global context within signals, albeit often at the cost of higher computational demands than CNNs.

For instance, transformer models originally designed for image recognition [13] have been adapted for high-density surface EMG gesture recognition, achieving accuracy that challenges or exceeds CNN benchmarks [14]. However, their large parameter counts and computational needs can limit their use in low-power prosthetic hardware, which led to the adoption of a compact 1D-CNN in this study; nevertheless, transformer-based methods remain an exciting avenue for future research.

A study employing the Hilbert-Huang Transform and deep neural networks achieved up to 98.5% accuracy, depending on the hand movement analyzed [6]. Naser et al. proposed a semi-supervised learning autoencoder framework that integrates a multilayer neural network for sEMG-based classification of five basic hand gestures, reporting an accuracy of 99.26%. However, the analysis was limited to simple hand gestures [7]. In another study, the GrabMyo dataset was utilized with ML algorithms applied for gesture classification, where a peak accuracy of 90.6% was achieved with a linear discriminant analysis-support vector machine combination [15]. Furthermore, Kim et al. developed an artificial neural network-based system capable of classifying seven hand gestures and three grasp types, reporting an accuracy of 97.49% but requiring extensive training sessions, which may have potentially skewed the reported outcomes [16]. As such, the persistent misclassification of hand gestures emphasizes the need to refine feature extraction techniques.

In the context of this study, the Ninapro dataset emerged as a promising tool that provides a comprehensive multi-channel EMG repository under standardized conditions [3,17], along with broad coverage of diverse hand gestures, which helps facilitate benchmarking and improve overall control algorithms [3,18]. Atzori et al. used a parallel-windows feature extractor to extract signal features from the Ninapro DB1–5 datasets, achieving sub-80% classification accuracy with a Random Forest classifier [19]. Using Ninapro DB5, Wang et al. combined CNNs, long short-term memory (LSTM), and convolutional block attention modules to achieve a 92.15% accuracy in hand gesture recognition [20]. Emima et al. proposed a multi-scale 1D–CNN framework, where accuracies of 93.4%, 92.8%, 91.3%, and 94.1% across Ninapro DB1, DB2, DB5, and DB7 were achieved respectively. Nonetheless, the computational complexity of the presented models poses significant challenges to future real-time deployment in low-power prosthetic devices.

Despite the progress made in sEMG-based hand gesture recognition, several challenges remain. The common presence of both noise and artifacts in sEMG signals poses significant obstacles to achieving reliable and validated classification results. In addition, generalizing models across varying subjects and environments remains a critical issue. Despite deep learning models such as CNNs and RNNs achieving high performance on specific datasets, performance commonly degrades when applied to new individuals or under varying conditions. Furthermore, an ongoing challenge persists in the trade-off between model complexity and interpretability, particularly in the context of medical applications, where transparency plays a key role in decision-making.

Previous studies have primarily focused on hand-gesture movements without specifically addressing individual finger movements, which are fundamental to hand gestures. From a clinical perspective, reliably decoding individual finger movements is paramount for restoring natural dexterity and allowing individuals with upper limb loss to perform a range of daily activities that require fine motor skills, including typing, operating tools, or grasping small objects. Moreover, existing research has utilized entire datasets while reporting overall accuracy yet failing to detail the sensitivity and specificity of each finger movement. These approaches overlook the possibility that some movements may be well recognized while others may not be. Therefore, this study focuses on the most complex movements, those of the fingers, to provide a comprehensive analysis of each movement. To address existing gaps in literature, we propose a tailored 1D-CNN approach for fine-resolution finger movement recognition. The distinction of our proposed method from existing deep learning approaches lies in an optimized balance between deep feature extraction and computational efficiency, achieved by pairing high-resolution signal preprocessing techniques with a streamlined 1D architecture. This will contribute to improving future recognition systems for prosthetic design and optimizing overall progress capabilities.

Research has mainly focused on classifying individual finger movements, not whole hand gestures. For example, Tenore et al. [21] achieved over 98% accuracy in decoding 12 finger motions using surface EMG and neural networks with five participants. Kanitz et al. [22] used linear discriminant analysis and support vector machines on eight finger primitives with one participant, reaching 97.7% accuracy. Al-Timemy et al. [23] combined features with discriminant analysis to classify various finger movements in ten intact-limbed and six amputee subjects. Malesevic et al. [24] introduced a model for real-time finger movement decoding for prosthetics. These studies show that finger classification is possible but rely on simple features and small groups, and report mainly overall accuracy without detailed per-movement analysis.

This study examines nine finger movements in a larger cohort of 40 subjects from the NinaPro DB2 dataset. It provides a systematic analysis of sensitivity, specificity, precision, and F1-score for each movement, addressing the clinical need for reliable finger movement decoding to restore dexterity and perform daily activities. Unlike prior work focused on overall accuracy, our approach uses a 1D-CNN with high-resolution preprocessing, offering detailed per-movement diagnostics. This method advances analytical depth, cohort size, and diagnostic granularity, aiming to improve prosthetic design and hand function recognition.

## 2. Methods

The approach proposed in this paper is illustrated in Fig 1. It begins with data as input, applies pre-processing techniques, and concludes with the classification of nine finger movements. Each corresponding subsection details the sequence of the methodology adopted.

**Fig 1:**
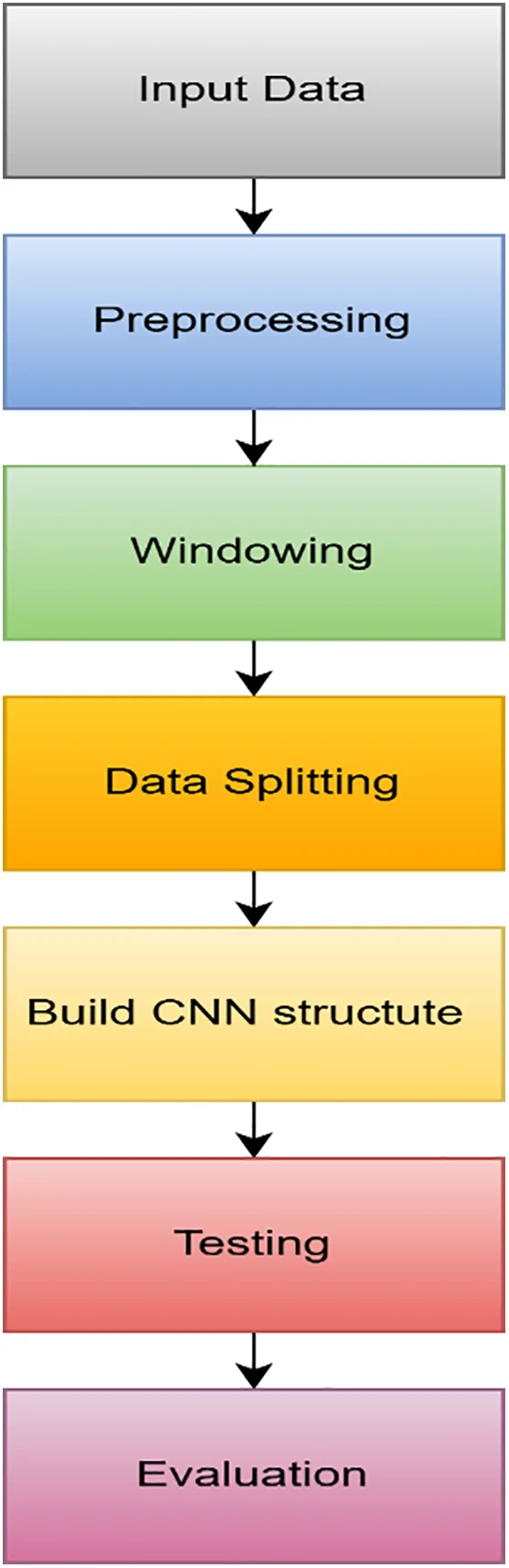
Overview of the proposed methodology pipeline.

### 2.1 Data Acquisition

Selecting an sEMG dataset is critical to ensure effective training and evaluation of hand gesture classifiers, as model accuracy and generalizability can vary widely from one dataset to another. The NinaPro DB2 dataset, comprising recordings from 40 participants performing 49 different hand movements using 12 surface electrodes, is a widely recognized benchmark in the prosthetic field [3]. The NinaPro DB2 dataset additionally provides inertial, kinematic, and force data to supplement comprehensive analysis. While other publicly accessible EMG datasets cover diverse demographics and amputee populations, NinaPro DB2 was determined to be the best fit.

Among the range of movement types offered in DB2, this study focuses on D movements, which primarily involve finger movements. A total of nine finger movements from the available data were included, each displaying the EMG waveform and corresponding to a color code (Fig 2).

**Fig 2:**
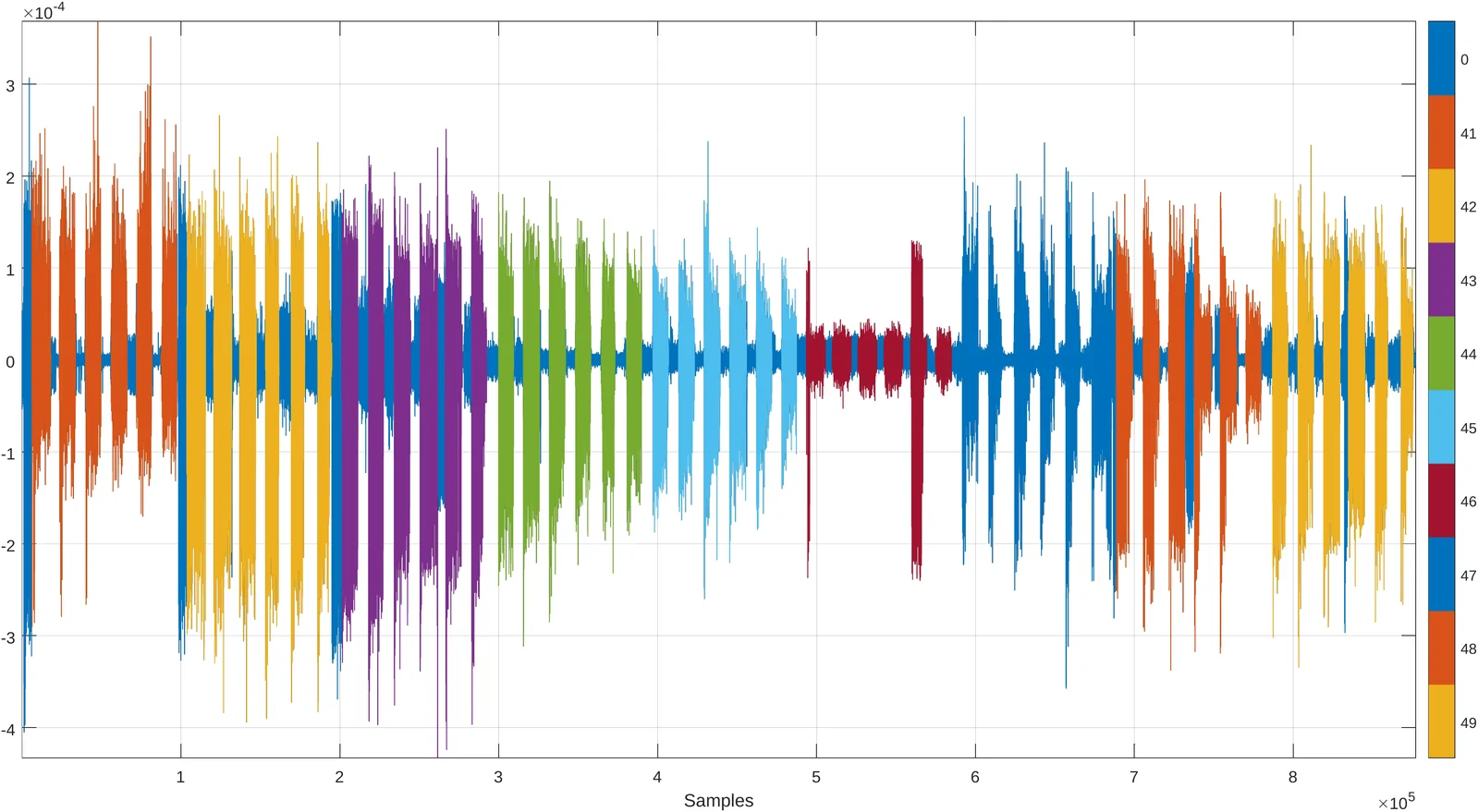
Color-coded EMG signal waveforms corresponding to each of the nine finger movements.

### 2.2 Pre-Processing

Several preprocessing steps have been taken to enhance sEMG signals. A band-pass filter (20–500 Hz) was applied to preserve EMG energy while attenuating unwanted frequencies, and a notch filter (50–60 Hz) was applied to suppress power-line interference [25,26]. The corresponding illustration, Fig 3, shows the pre-processing steps in this paper.

**Fig 3:**
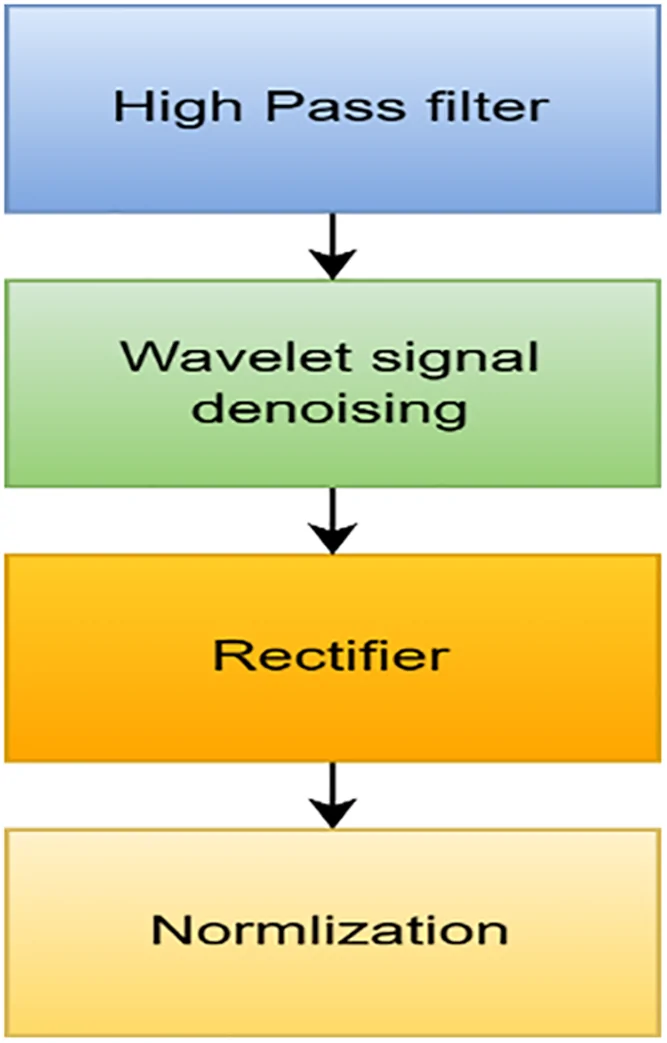
Demonstration of the sEMG signal denoising process.

#### 2.2.1 *Denoising-based Wavelet.*

Wavelet-based denoising is a widely adopted method that decomposes EMG signals into multiple frequency components through wavelet transformations. Since signal noise typically involves high-frequency components, a threshold is applied: small coefficients are either reduced or set to zero, while significant signal coefficients are preserved [27–29]. The denoised and filtered signal is further reconstructed via an inverse wavelet transform. In this stage, we specified the mother wavelet (Daubechies 4) and the exact number of decomposition levels (5) applied before empirical Bayesian thresholding [28,30]. Fig. 4 presents the original and denoised sEMG signals after wavelet-based and Bayesian denoising.

**Fig 4:**
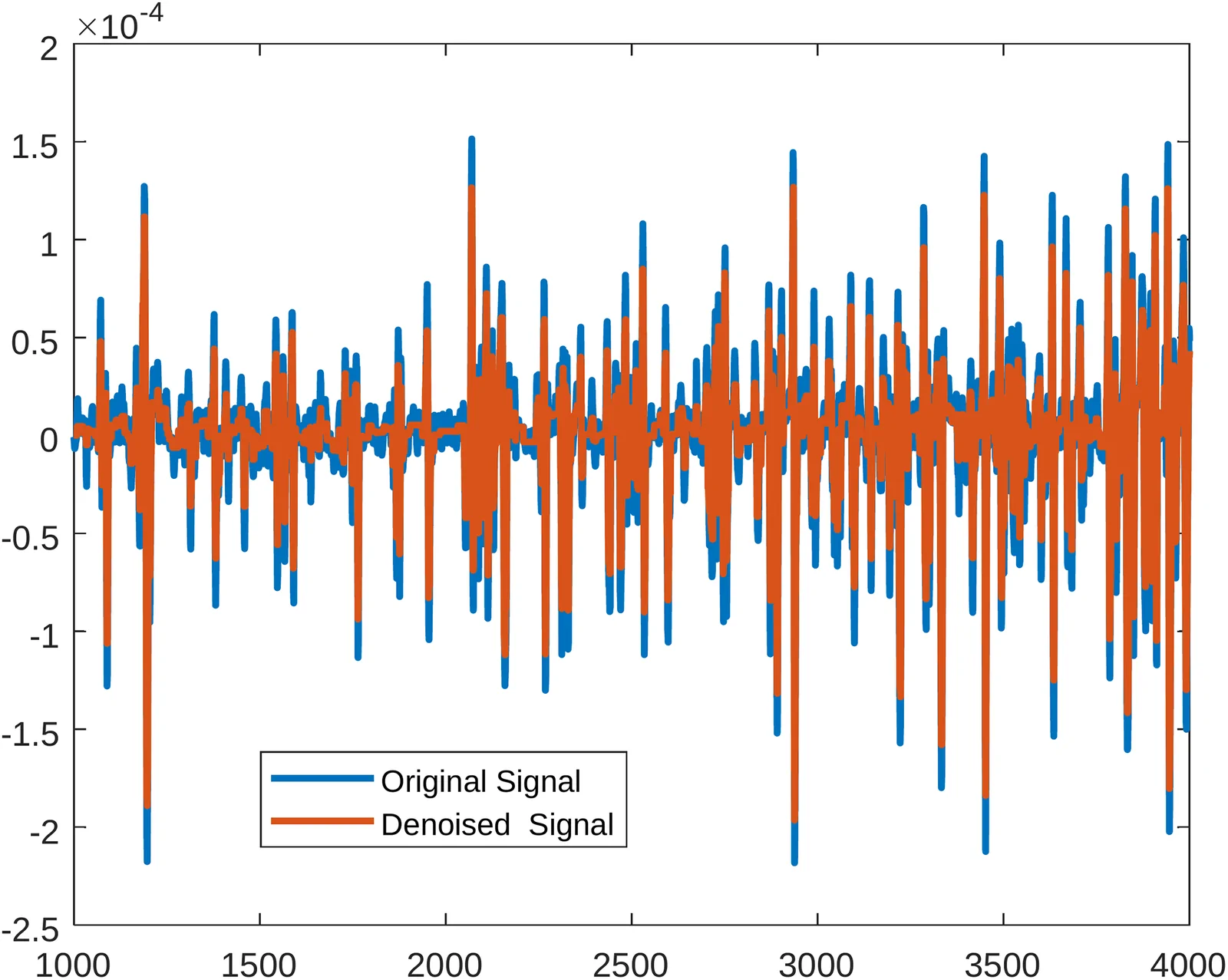
Comparison between original and denoised sEMG signals after wavelet-based and Bayesian denoising techniques.

#### 2.2.2 *Rectification.*

Rectification is a commonly applied technique to EMG signals following band-pass filtering, where bipolar waveforms are converted into unipolar signals by extracting absolute values of the signal at designated time points. Full-wave rectification was applied, converting negative amplitudes to positive to ensure that all muscle activation energy is represented by positive magnitudes [31]. Fig 5 illustrates the rectified EMG signal.

**Fig 5:**
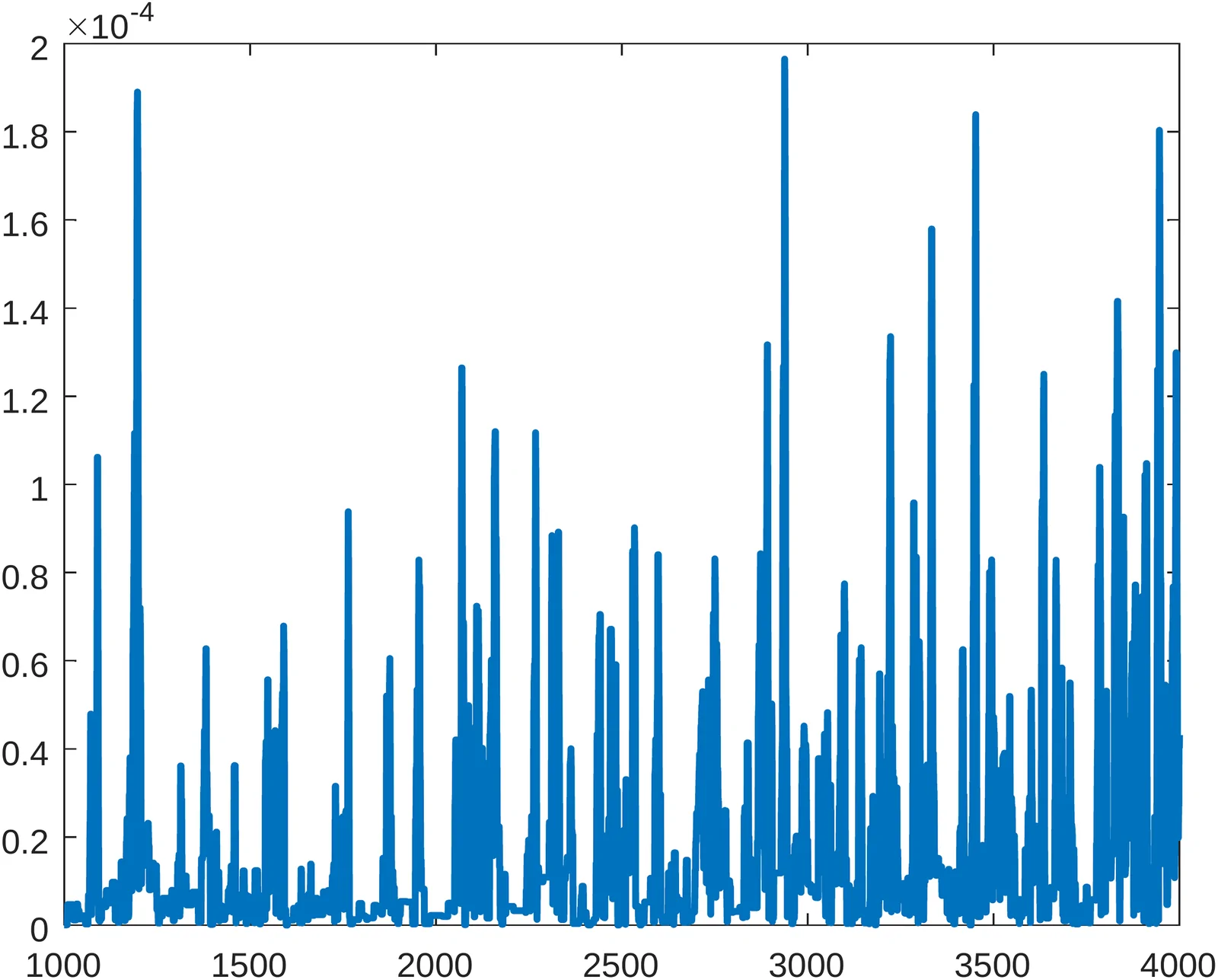
Full-wave rectified sEMG signals.

#### 2.2.3 *Normalization.*

The normalization step is applied to maintain consistency in EMG signal amplitudes across channels, subjects, and recording sessions. In this paper, we strictly applied the Z-score standardization method (zero mean, unit variance) calculated independently for each of the 12 channels (per-channel normalization) to prevent higher-amplitude channels from dominating the feature space during 1D-CNN training by scaling signals relative to a reference contraction, enabling standardized interpretation of signal strength across subjects in a dataset [32]. Z-scores are used because they are less affected by isolated high-amplitude outliers. Fig 6 highlights the normalized signal.


$$\begin{array}{c} x_{norm}(t)=\frac{x_{rect}(t)-\mu }{\sigma } \end{array}$$

**Fig 6:**
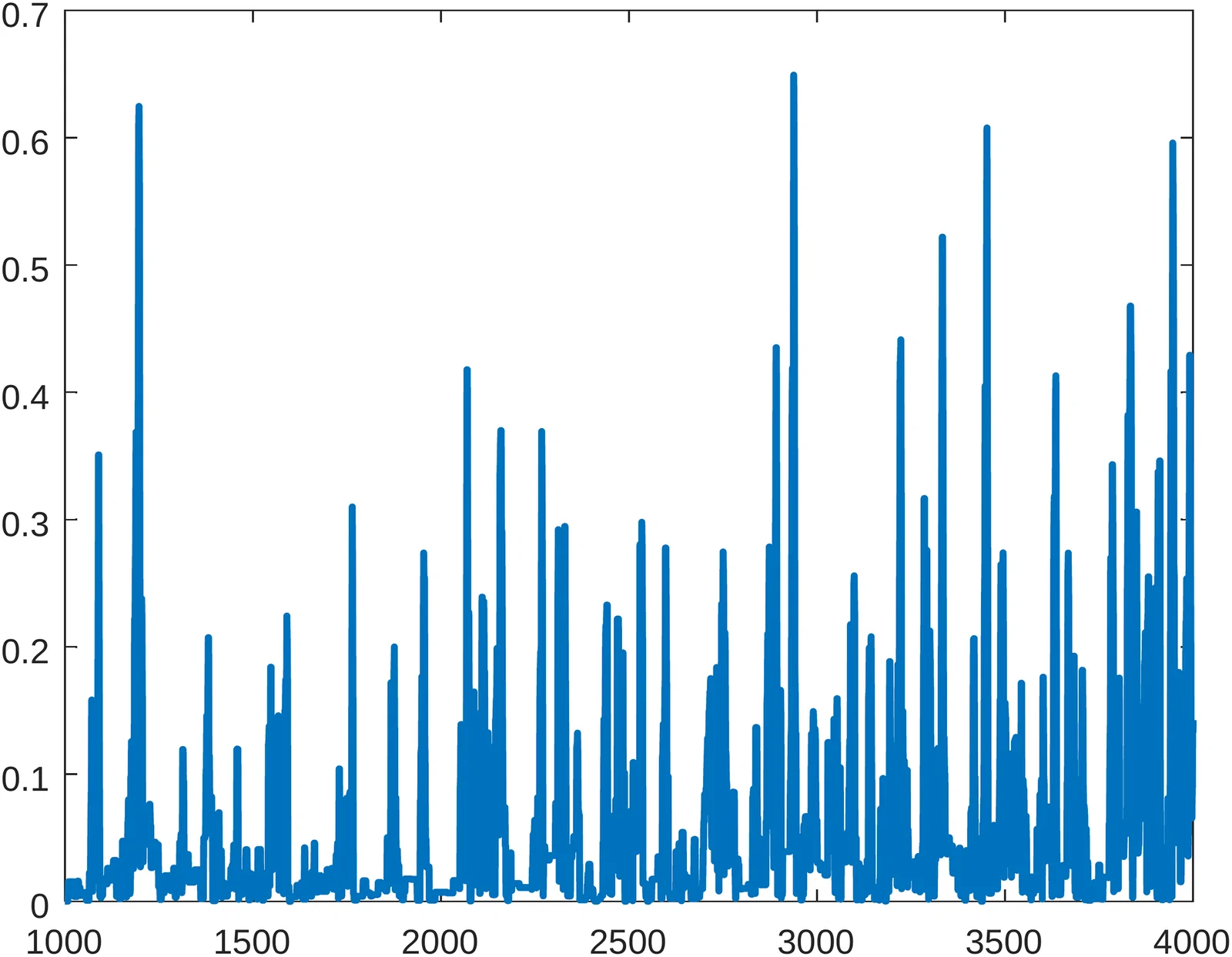
Normalized sEMG signal after applying z-score standardization.

Where:

- μ is the mean of the training signal
- σ is the standard deviation of the training signal

### 2.3 Windowing

A necessary step is implementing sliding-window segmentation to divide continuous EMG into overlapping short windows, aiming to achieve high hand gesture recognition accuracy. Each window serves as a sample for classification to capture temporal patterns of muscle activation, aiming to achieve accurate gesture recognition. Finding an optimal balance between information content and responsiveness is paramount: selecting a long window introduces potential phases or transitions, whereas a short window may fail to capture the full gesture pattern [33].

A 400 ms window with 50% overlap was selected to fully capture slower and more complex gestures. Despite a 400 ms window exceeding the typical 150–250 ms recommendation for a real-time myoelectric window, it was selected for this offline study because longer windows yield superior recognition for refined and coordinated finger movements with distributed EMG patterns. Small windows, on the other hand, tend to have difficulty distinguishing similar movements. This decision stemmed from qualitative rather than systematic evaluation, and no accuracy-latency trade-off analysis was performed. Exploring various window lengths (e.g., 150–400 ms) to optimize both performance and response time presents promising grounds for future real-time research [34]. The illustration below, Fig 7, presents the optimized window selection.

**Fig 7:**
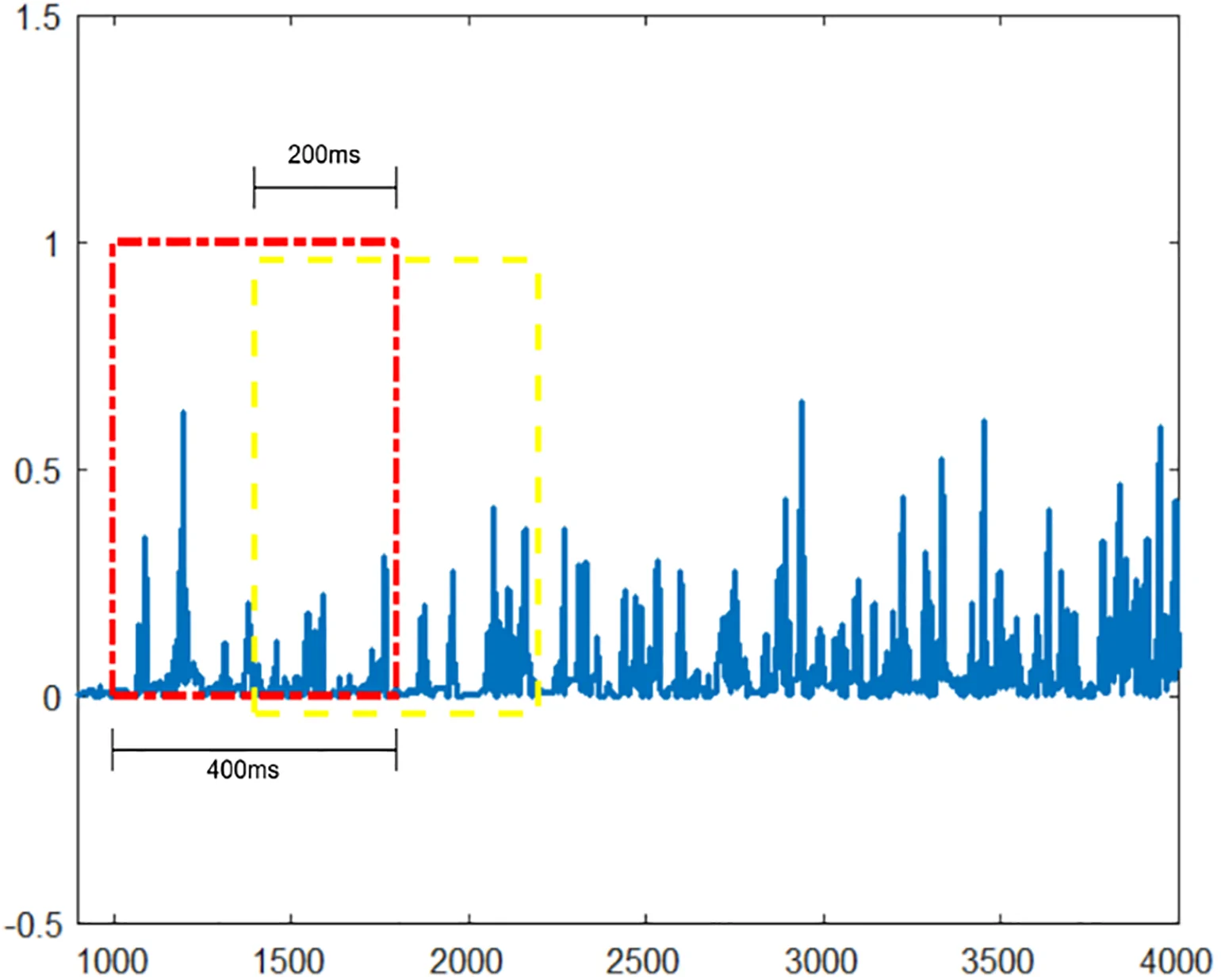
The optimized window size with overlapping process.

### 2.4 Data Splitting

To avoid temporal correlation, this study utilized repetitions 2 and 5 from the NinaPro DB2. The model was trained and tested on the same individual across different repetitions, commonly referred to as within-subject evaluation, as opposed to cross-subject evaluation, which uses data from multiple individuals. Recent literature suggests partitioning the data into about 60–70% for training, 10–20% for validation, and 10–30% for testing. Selecting repetitions 2 and 5 keeps assessment bias risk to a minimum by preventing the model from memorizing data patterns.

### 2.5 Convolutional Neural Network (CNN)

Convolutional neural networks (CNNs) are widely used in EMG classification for their ability to extract deep features using optimal filters automatically. 1D-CNNs effectively process multichannel EMG signals over time by capturing both spatial and temporal features, including local motifs, thereby aiding hand gesture recognition. They are faster, simpler, and easier to train than RNNs such as LSTMs, offering a good balance of accuracy and efficiency, especially with refined regularization [35]. The 1D-CNN architecture starts with an input layer, followed by convolutional layers with filters and activation functions, a global pooling layer, a fully connected layer, a Softmax layer, and classification layers. It was trained with the Adam optimizer for 500 epochs and specific hyperparameters, demonstrating its effectiveness in EMG signal classification (Fig 8).

**Fig 8:**
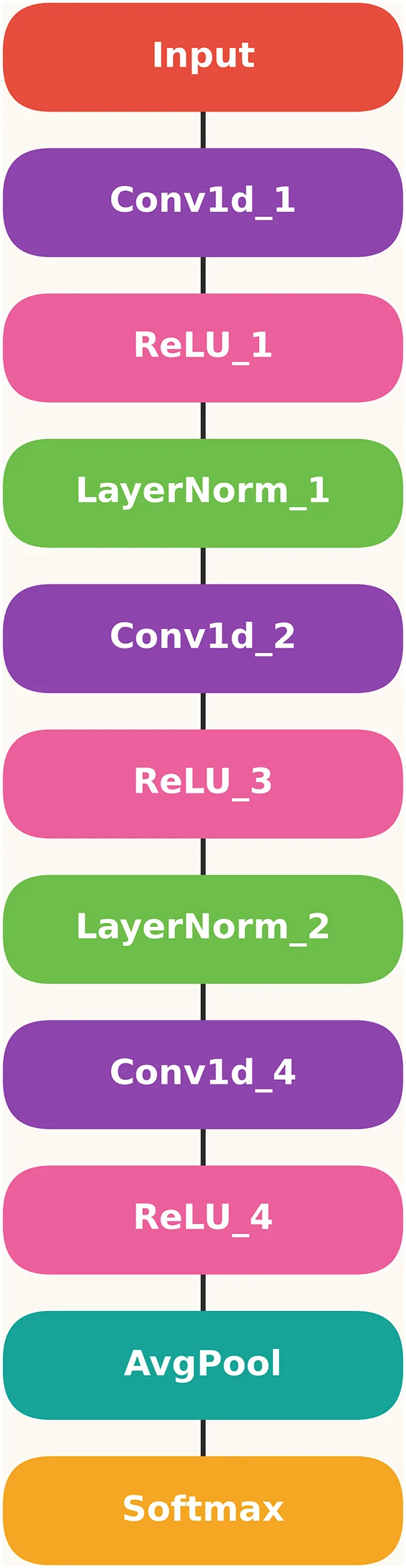
Schematic of proposed 1D–CNN model architecture.

For hyperparameter tuning, we empirically optimized all parameters. We conducted an iterative evaluation of different configurations, selecting the combination that yielded the most stable convergence on the validation set without oscillations. To prevent overfitting, we employed a multi-layered approach:

1. We applied L2 Regularization (weight decay) with a coefficient of 0.001 to penalize large weights.
2. We enforced dynamic data shuffling (Shuffle: every epoch) to prevent the network from memorizing sequence patterns.
3. We continuously monitored the validation loss through an independent validation set to ensure no generalization gap emerged between the training and testing phases.

xϵ $R^{CxT}$: input EMG segment with C = 12 channels, T = 800 time steps

- $w^{\left(l\right)}$ ϵ $R^{FxCxK}$: weights of the convolution layer *l*
- $y^{\left(l\right)}$: output feature map

$\begin{array}{c} \overset{l}{\underset{f,t}{y}}=\sum_{c=\text{1}}^{C}\sum_{k=\text{0}}^{K-\text{1}}\overset{\left(l\right)}{\underset{f,c,k}{w}}\cdot x_{c,t+k}+\overset{\left(l\right)}{\underset{f}{b}} \end{array}$

Where:

- *f*: filter index
- *k*: kernel index
- *K*: kernel size

## 3. Results

The model is trained using the previous settings mentioned in the methods section; the results are summarized in the confusion matrix and the receiver operating characteristic (ROC) curve in Fig 10.

As shown in the confusion matrix in Fig 10a, the classifier’s overall accuracy across all movements was 90.1%. A total of 1,777 records were correctly classified, achieving a precision of 89.7% and a recall of 93.1% for the flexion of the little finger labeled “41”. Label “42” corresponds to the flexion of the ring finger and achieved 1,604 records that were correctly classified, with a precision of 90.2% and a recall of 87.6%. Label “43” represents flexion of the middle finger, which recorded 1,575 correct classifications, yielding a precision of 89.4% and a recall of 89.7%. Label “44”, representing flexion of the index finger, had a total of 1,514 correctly classified instances, with a precision of 88.5% and a recall of 90.6%. As for label “45”, a total of 1,452 records were correctly classified for the thumb abduction movement, with a precision of 90.8% and a recall of 89.8%. The thumb flexion movement (label “46”) achieved 1,820 correct classifications, with precision 91.9% and recall 91.6%. A total of 1,460 records were correctly classified for flexion of the index and little fingers in label “47”, with precision 88.2% and recall 90.3%. Flexion of the ring and middle fingers, in label “48”, had 1513 correct records, with a precision of 91.1% and a recall of 88.5%. For the final label, “49”, representing the flexion of the index finger and thumb, a total of 1,392 records were correctly classified, with a precision of 91% and a recall of 89.2%.

The metrics that have been demonstrated in this paper include the following:


$$\begin{array}{c} Recall=\frac{TP}{TP+FN} \end{array}$$

$$\begin{array}{c} Precision=\frac{TP}{TP+FP} \end{array}$$

$$\begin{array}{c} F\text{1}-score=\frac{\text{2}\times Recall\times Precision}{Recall+Precision} \end{array}$$

$$\begin{array}{c} Specificity=\frac{TN}{TN+FP} \end{array}$$

Where:

- *TP*: true positive classified cases
- *TN*: true negative classified cases
- *FP*: false positive classified cases
- *FN*: false negative classified cases.

Table 1 describes the obtained results, which are shown in the confusion matrix for all 9 finger movements. According to Table 1, thumb flexion achieved the highest precision, while index finger flexion had the lowest. In terms of recall, little finger flexion recorded the highest recall, while index and little finger flexion scored the lowest recall. For the F1 score, thumb flexion again achieved the highest value (0.916), while index and little finger flexion had the lowest value (0.888). Regarding specificity, index and thumb flexion had the highest value (0.989), while little finger flexion had the lowest (0.983).

**Table 1:** Results obtained for 9 different movement classes.

Class	TP	FP	FN	TN	Precision	Recall	F1-Score	Specificity
**41 – Little Finger flexion**	1777	203	132	11888	0.897	0.931	0.914	0.983
**42 – Ring Finger flexion**	1604	195	173	12028	0.892	0.903	0.897	0.984
**43 – Middle Finger flexion**	1575	173	175	12077	0.901	0.9	0.901	0.986
**44 – Index Finger flexion**	1514	196	167	12123	0.885	0.901	0.893	0.984
**45 – Thumb Abduction**	1452	148	175	12225	0.908	0.892	0.9	0.988
**46 – Thumb Flexion**	1820	158	177	11845	0.92	0.911	0.916	0.987
**47 – Index+Little Flexion**	1460	181	186	12173	0.89	0.887	0.888	0.985
**48 – Ring+Middle Flexion**	1513	178	132	12177	0.895	0.92	0.907	0.986
**49 – Index+Thumb Flexion**	1392	137	168	12303	0.91	0.892	0.901	0.989

Fig 9 illustrates the histogram analysis of sensitivity, specificity, precision, and F1-score across all classes.

**Fig 9:**
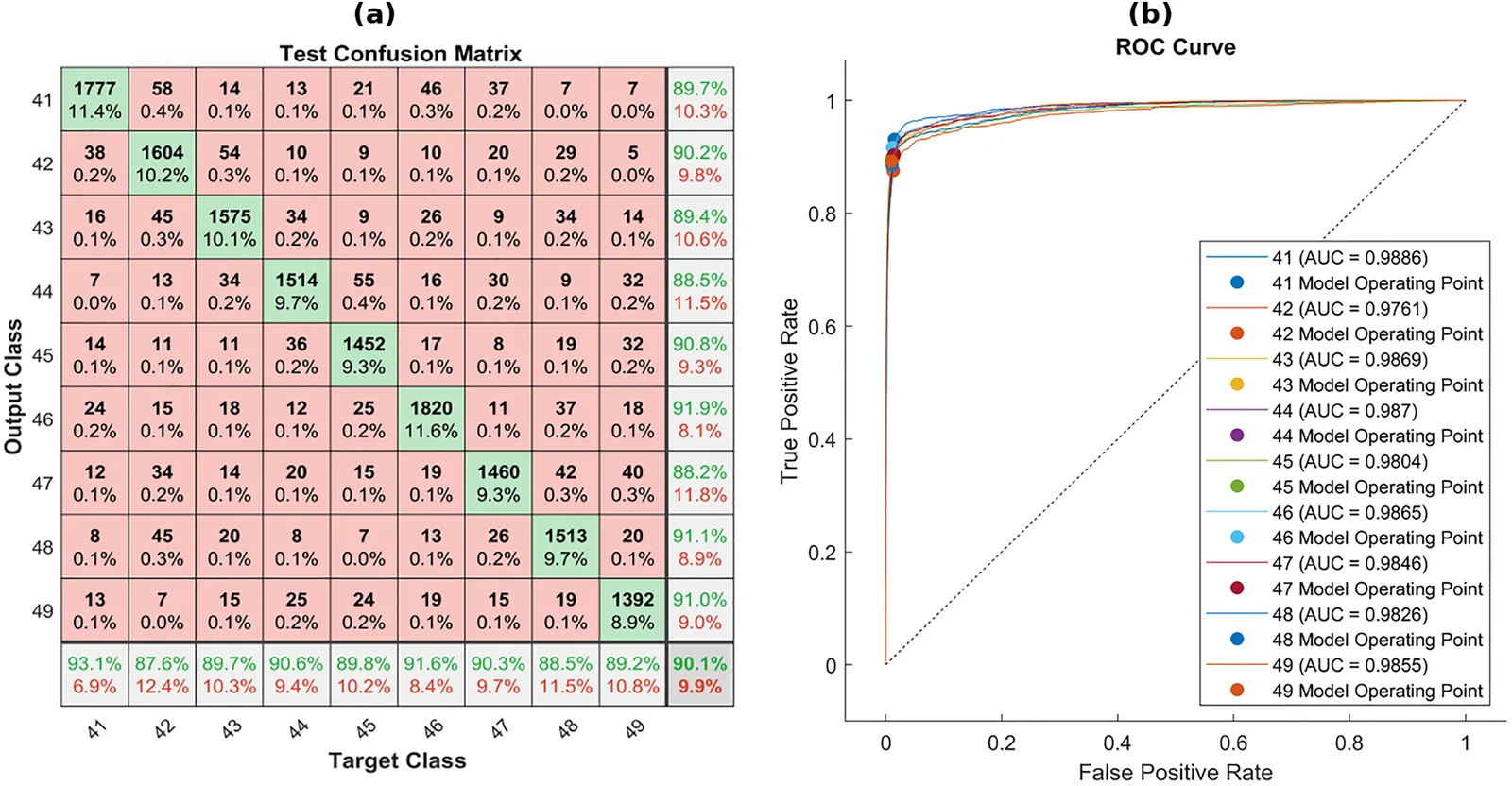
(a) The obtained confusion matrix, (b) the resultant ROC curve.

To statistically validate the robustness of this performance, a 95% Confidence Interval (CI) was calculated using the normal approximation interval for binomial proportions. Based on the total test sample size, the 95% CI for the overall accuracy is strictly bounded at [89.63%, 90.57%]. This exceedingly narrow margin of error (± 0.47%) indicates high statistical reliability of the proposed 1D-CNN model across the tested iterations. An illustration of CI calculations is shown in Fig 10, Fig 11.

**Fig 10:**
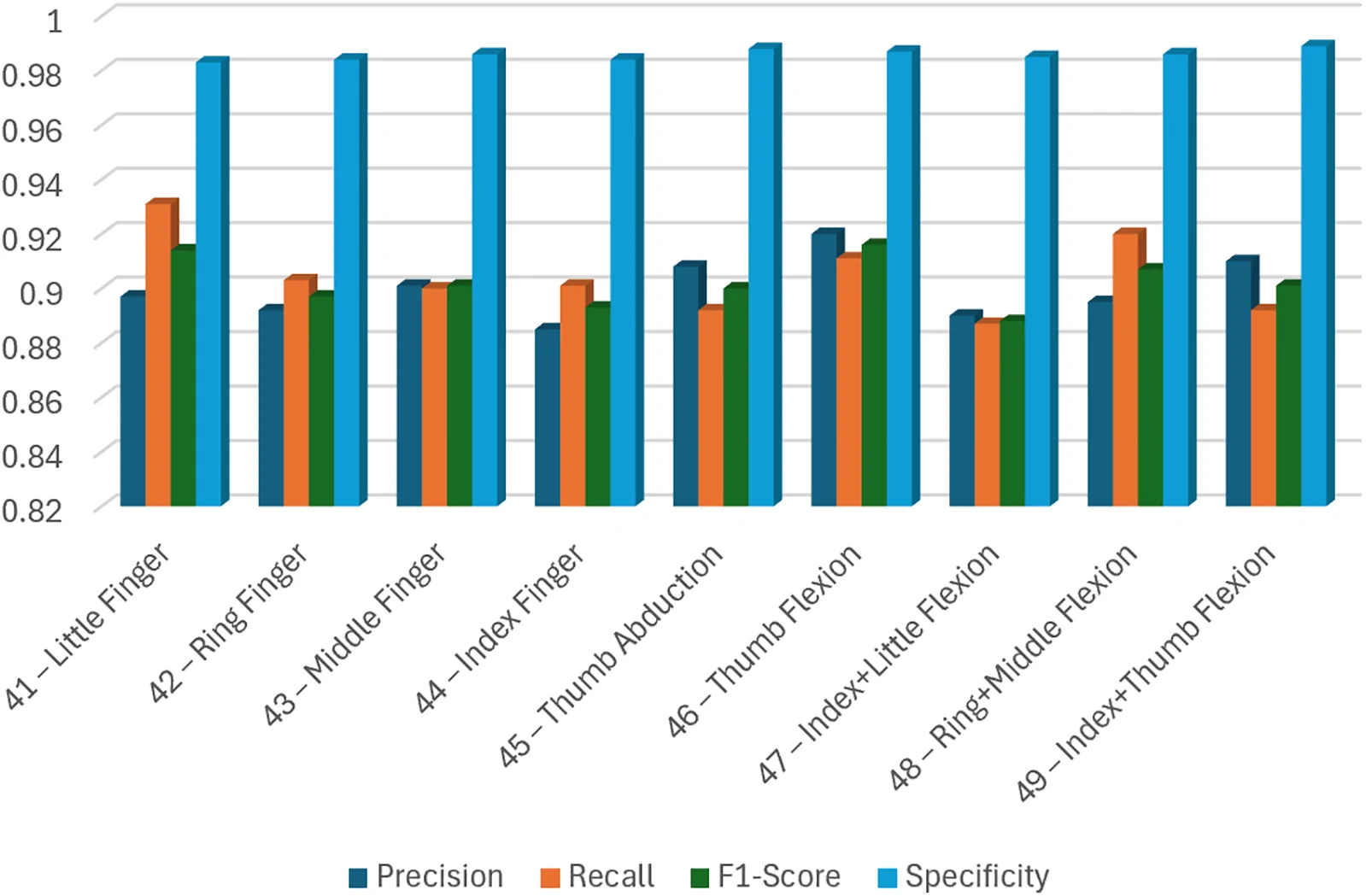
Histogram analysis summarizing sensitivity, specificity, precision, and F1-score for each classified finger movement.

**Fig 11:**
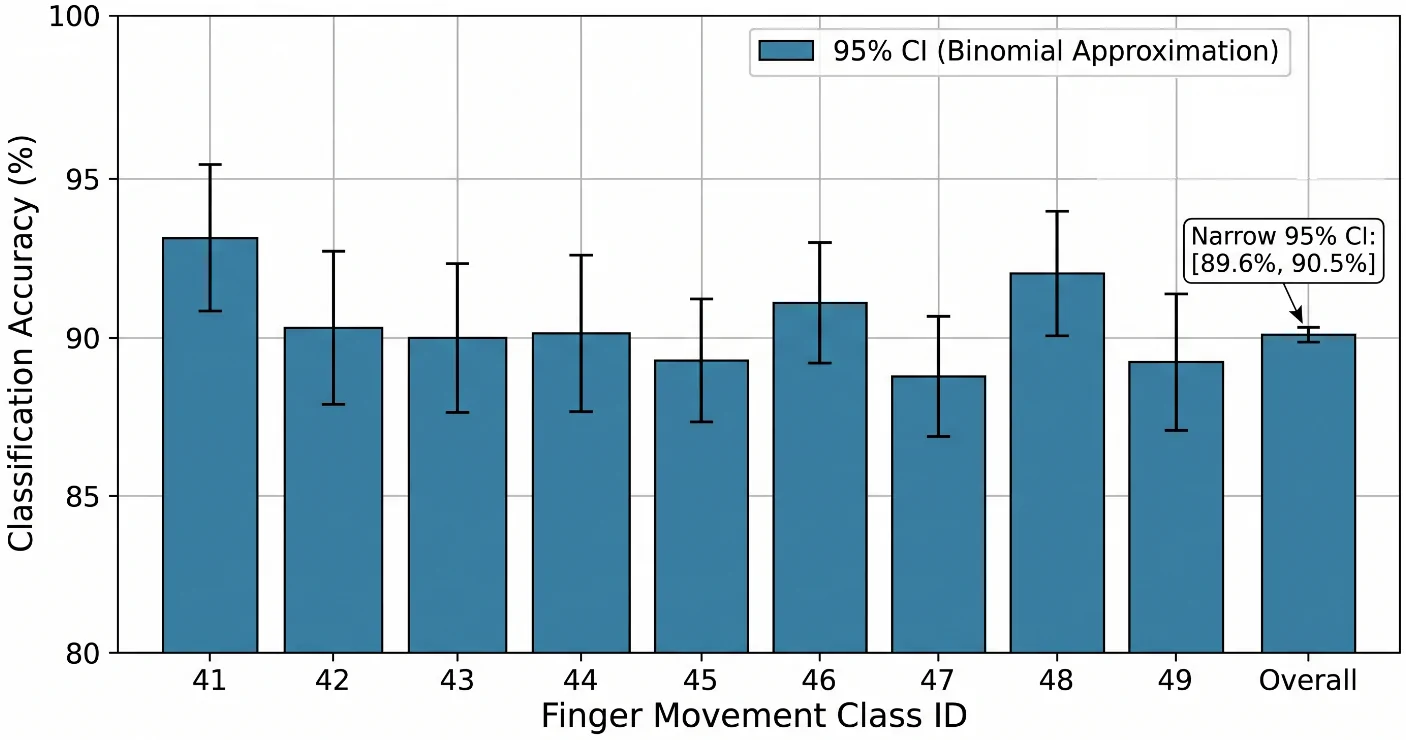
CI calculations across 10 different movement classes.

## 4. Discussion

This study addresses surface electromyography (sEMG)-based prosthetic control by targeting the classification of individual finger movements. This area has received less attention than whole-hand gesture recognition. While a limited number of previous studies have explored individual finger movements using classical time-domain approaches [30–33], deep learning methods for this task remain comparatively underexplored. The proposed custom 1D-CNN model reached an accuracy of 90.1%, with sensitivity and specificity analyzed for each of the nine finger movement classes.

Most recent studies have focused on classifying different hand gestures and have reported mainly overall classification accuracy. For example, Naser et al. [7] achieved 99.26% accuracy for five basic hand gestures using semi-supervised autoencoders, while Kim et al. [16] reported 97.49% accuracy for ten hand movements including grasp tasks. Although these results are promising, the proposed models generally require extensive training and may have limited ability to generalize across different users. Furthermore, these studies reported only overall accuracy without reporting class-specific performance metrics, such as sensitivity and specificity.

Compared with studies that investigated individual finger movement classification, Tenore et al. [30] and Kanitz et al. [31] reported higher classification accuracies (above 97%). However, both studies were conducted on very small, single-session datasets (five and one subjects, respectively) and used classical time-domain features. Such designs are more susceptible to overfitting and may not generalize to larger and more diverse populations. Al-Timemy et al. [23] have used time-domain autoregressive features with discriminant analysis classifiers to classify different finger movements for ten intact-limbed and six below-elbow amputee persons. Another study by Malesevic et al. [33] used a hierarchical hidden Markov model aimed at real-time control, which is a methodologically closer comparator; unlike the present study, it used EMG signals from five able-bodied subjects and three amputees. Nevertheless, neither study reported detailed class-wise performance metrics, such as sensitivity, specificity and F1, across as many as nine finger movements in a 40-subject cohort. These comparisons indicate that, although the present model doesn’t outperform previous studies in accuracy, it offers a competitive performance with more comprehensive evaluation in a larger dataset.

This study did not directly compare the proposed method with conventional time-domain (TD) features using a linear discriminant analysis (LDA) classifier. Prior literature applying TD-feature-based classifiers (including LDA and related discriminant-analysis variants) to individual and combined finger movement classification [31,32] provides an indirect benchmark and suggests that such approaches are viable for this task. Khushaba et al. [36] used TD features with LDA for dimensionality reduction and a SVM classifier, further advanced using a Bayesian fusion voting approach. Using two sEMG channels, their method achieved 92% offline classification accuracy for ten classes of individual and combined finger movements. Al-Timemy et al. [23] also reported comparable classification performance using time-domain autoregressive (TD-AR) features with LDA from ten intact-limbed and six amputee persons. The results showed that TD-LDA methods can reach accuracies within a range comparable to the 90.1% achieved by the proposed 1D-CNN in the current study. However, the comparison should be interpreted with caution because of differences in cohort size, electrode configuration, number of EMG channels, and movement sets. We were not able to re-implement and re-evaluate a TD + LDA baseline on the same NinaPro DB2 subset used here within the scope of this study, and we consider this a specific, addressable limitation; a matched TD + LDA baseline evaluated under identical data splits would provide the clearest evidence of the benefit, if any, offered by the proposed 1D-CNN over conventional myoelectric pattern recognition and is planned as a direct extension of this work.

In contrast, this paper provides an in-depth analysis across nine distinct finger movements using the standardized and comprehensive NinaPro dataset, contributing to a more robust recognition capability. Although Emima et al. [19] reported accuracies ranging from 91% to 94% using multi-scale 1D-CNN models on NinaPro DB1–DB7 datasets, their study focused on hand gesture recognition rather than classification, and they did not evaluate real-time feasibility.

The proposed 1D-CNN achieves a practical balance between classification accuracy and computational complexity. Compared with a hybrid CNN-LSTM-CBAM model proposed by Wang et al. [18], which reached 92.15% accuracy, our approach delivers competitive performance with a substantially simpler architecture and reduced computational requirements. This makes it a promising model for eventual embedded or real-time deployment. However, it is important to mention that the current study evaluated the model only offline, and no real-time or latency testing was performed. Therefore, its suitability for real-time control should be investigated in future work.

Furthermore, the proposed approach incorporates signal preprocessing and high-resolution feature conditioning to enhance EMG signal quality, which could provide more stable and reliable classification performance, particularly in the presence of signal noise and inter-user differences. These issues were also highlighted by Atzori et al. [17] and other researchers; their studies, which primarily used random forest and other conventional machine learning classifiers, reported lower classification performance, typically below 80% accuracy.

We compared our results to prior studies that used the same NinaPro DB2 dataset. Geng et al. [37] used an EMG-image-based CNN architecture to classify 49 gestures, achieving 76.1% accuracy. Ding et al. [38] used a parallel multiscale convolutional architecture, achieving 78.9% accuracy across 17 gestures from NinaPro DB2. A two-stream residual network with an attention mechanism was used for gesture recognition, achieving 88.25% accuracy [39]. In another study, a transfer-learning CNN framework was applied to NinaPro DB2 exercise B (17 hand movement classes), improving accuracy from 81.4% to 82.9% through subject-transfer learning [40]. Compared with these DB2-based studies, the proposed model achieves a higher overall accuracy (90.1%) and performs a more fine-grained classification of individual and combined finger movements rather than whole-hand gestures.

These findings are relevant to the development of the next generation of intelligent prosthetic devices, in which accurate classification opens possibilities for more natural and accessible human-machine interface interaction. The results of this study should be interpreted as technical feasibility rather than clinical benefit. The offline accuracy reported in this study indicates that fine-resolution finger movement classification is possible from available EMG data, but further validation in real-time and clinical settings is required. Table 2 summarizes a comparison between the present study and the previous literature.

**Table 2:** Literature comparison of sEMG-based gesture and finger-movement recognition studies.

Study	Dataset used	Methodology	Movement types	Accuracy	Granularity	Limitations
**Naser et al. [7]**	Custom	Semi-supervised autoencoders	5 hand gestures	99.26%	Low	Basic gestures only
**Kim et al. [16]**	Custom	ANN	10 gestures incl. grasps	97.49%	Medium	Requires extensive training sessions
**GrabMyo [15]**	GrabMyo	LDA + SVM	5 gestures	90.6%	Low	Limited gesture scope
**Emima et al. [25]**	NinaPro DB1–7	Multi-scale 1D-CNN	Full gesture sets	91–94%	Medium	High computational complexity
**Wang et al. [20]**	NinaPro DB5	CNN + LSTM + CBAM	Hand gestures	92.15%	Medium	Complex model architecture
**Atzori et al. [19]**	NinaPro DB1–5	Feature extractor + RF	Hand gestures	<80%	Low	Lower accuracy, traditional ML approach
**Tenore et al. [21]**	Custom (5 subjects)	TD features + ANN	12 finger movements	>98%	High	Small, single-session cohort
**Kanitz et al. [22]**	Custom (1 subject)	LDA/ SVM	8 finger motion primitives	97.7%	High	Single-subject; not generalizable
**Al-Timemy et al. [23]**	Custom (16 subjects)	TD-AR features + OFNDA + LDA	Individual + combined finger movements	High (not directly comparable)	High	Different electrode set; amputee and intact-limbed mix
**Malesevic et al. [24]**	Custom	VAR hierarchical HMM	Individual finger movements	Comparable to best classifier tested	High	Small cohort; real-time focus, limited diagnostic breakdown
**This study**	***NinaPro DB2 (40 subjects)***	***1D-CNN***	***9 individual + combined finger movements***	***90.1% (95% CI 89.6–90.6%)***	***High***	***Offline, within-subject only; no amputee or real-time validation; no TD + LDA baseline.***

Direct numerical comparison across the studies summarized in Table 2 should be interpreted cautiously: the compared studies differ in dataset (custom laboratory recordings vs. NinaPro), electrode count and placement, movement granularity (whole-hand gestures vs. individual/combined finger movements), cohort size and composition (intact-limbed vs. amputee participants), and evaluation protocol (within-subject vs. across-subject). None of the reported accuracy differences in Table 2 have been tested for statistical significance across studies, as the underlying per-subject or per-fold data from other published works were not available to us; the comparison is therefore intended to contextualize, rather than statistically rank, the performance of the proposed method.

## 5. Limitations

Several key limitations are found within this presented study. First, all evaluation was performed in an offline environment and within a single publicly available dataset (NinaPro DB2) that is collected from intact-limbed participants via a fixed electrode configuration. No amputee data were used, and no demonstration of generalization to amputee populations, alternative electrode placements, or other acquisition hardware was performed. Second, while within-subject was selected as the evaluation strategy in this study, the method fails to assess the model’s performance on a new user without subject-specific calibration data. Third, no real-time or embedded-hardware testing was conducted. The reported accuracy, along with the model’s relatively compact architecture, presents favorable indicators for eventual real-time use; however, processing latency, power consumption, and closed-loop usability with an actual prosthetic device remain key areas of evaluation. Fourth, the 400 ms analysis window exceeds the typical recommendation for low-latency myoelectric control [28], and we did not perform a systematic accuracy-versus-latency sweep across window lengths. Fifth, while we reported 95% CI for the pooled, within-subject accuracy of each movement class analyzed, we did not conduct any between-subject variability analysis, where classification performance may differ meaningfully across individual participants; a formal per-subject or mixed-effects statistical analysis was not conducted. Finally, a formal ablation study isolating the contribution of the normalization step was not performed within our framework. We view each of the aforementioned points as a concrete and addressable direction for follow-up work rather than a fundamental barrier to the feasibility arguments advanced in this study.

## 6. Conclusion

This study aimed to introduce a comprehensive framework to enhance prosthetic hand and robotic control applications through accurate classification of finger movements. Using advanced signal processing techniques for data preparation along with a 1D-CNN architecture for classification, this study explores a scalable range of high-resolution finger gestures including both individual finger movements and combined movements. The evaluation was conducted offline on the publicly available NinaPro DB2 dataset of intact-limbed participants. The proposed model architecture achieved high classification performance across the nine movement classes evaluated, with precision ranging from 0.89 to 0.92, recall from 0.89 to 0.93, and F1-scores consistently above 0.88, with 95% CIs reported for per-class accuracy.

Compared with other relevant work in EMG-based gesture recognition, where much of the focus is placed on broader movements with limited finger-level resolution, this study provides a significant improvement in both granularity and accuracy. Previous studies using traditional learning algorithms such as SVMs or k-nearest neighbors have reported average F1-scores in the range of 0.75–0.85 for basic hand gestures. In contrast, the deep learning-based framework adopted in this study captures subtle differences between similar movements (e.g., index + little finger flexion versus ring + middle finger flexion) while achieving relatively superior performance across all metrics evaluated.

The hypothesized effectiveness of combining advanced signal preprocessing with deep learning for EMG-based prosthetic control applications is confirmed through the results presented in this study. Given the limitations of this framework, including offline operation, within-subject evaluation, and an intact-limbed population, we present these results as evidence of technical feasibility rather than a validated clinical or real-time solution. This framework aims to offer amputees enhanced functional control and dexterity for their prosthetic devices, where the high accuracy and specificity found in the proposed model warrant its position as a candidate approach worth further development toward real-time prosthetic hand systems.

Future work should involve real-time deployment, integration with embedded systems and prosthetic devices, and data from amputee subjects to effectively assess the model’s clinical applicability and overall generalization. Amputee validation is regarded as a prerequisite before any claim for clinical translation can be supported due to EMG characteristics exhibiting significant differences for residual limbs relative to intact-limbed participants.
